# Cord Blood SARS-CoV-2 IgG Antibodies and Their Association With Maternal Immunity and Neonatal Outcomes

**DOI:** 10.3389/fped.2022.883185

**Published:** 2022-06-29

**Authors:** Addy Cecilia Helguera-Repetto, Isabel Villegas-Mota, Guadalupe Itzel Arredondo-Pulido, Jorge Arturo Cardona–Pérez, Moises León-Juárez, Maria Antonieta Rivera-Rueda, Gabriela Arreola-Ramírez, Paloma Mateu-Rogell, Sandra Acevedo-Gallegos, Gloria Elena López-Navarrete, María Yolotzin Valdespino-Vázquez, Guadalupe Martínez-Salazar, Mario Rodríguez-Bosch, Irma Alejandra Coronado-Zarco, María del Rosario Castillo-Gutiérrez, Carlos Alberto Cuevas-Jiménez, Elsa Romelia Moreno-Verduzco, Salvador Espino-y-Sosa, Manuel Cortés-Bonilla, Claudine Irles

**Affiliations:** ^1^Department of Immunobiochemistry, Instituto Nacional de Perinatología, Mexico City, Mexico; ^2^Department of Infectious Diseases and Epidemiology, Instituto Nacional de Perinatología, Mexico City, Mexico; ^3^General Director, Instituto Nacional de Perinatología, Mexico City, Mexico; ^4^Neonatal Intensive Care Unit, Instituto Nacional de Perinatología, Mexico City, Mexico; ^5^Department of Pediatric Follow-Up, Instituto Nacional de Perinatología, Mexico City, Mexico; ^6^Clinical Research Subdivision, Instituto Nacional de Perinatología, Mexico City, Mexico; ^7^Department of Materno-Fetal Medicine, Instituto Nacional de Perinatología, Mexico City, Mexico; ^8^Department of Pathological Anatomy, Instituto Nacional de Perinatología, Mexico City, Mexico; ^9^Gynecology and Obstetrics Subdivision, Instituto Nacional de Perinatología, Mexico City, Mexico; ^10^Neonatalogy Subdivision, Instituto Nacional de Perinatología, Mexico City, Mexico; ^11^Laboratorio Lapi S.A. de C.V., Mexico City, Mexico; ^12^Diagnostic Auxiliary Services, Instituto Nacional de Perinatología, Mexico City, Mexico; ^13^Medical Division, Instituto Nacional de Perinatología, Mexico City, Mexico; ^14^Department of Physiology and Cellular Development, Instituto Nacional de Perinatología, Mexico City, Mexico

**Keywords:** pregnancy, COVID-19, newborn, seropositivity, SARS-CoV-2 antibodies

## Abstract

Passive transplacental immunity is crucial for neonatal protection from infections. Data on the correlation between neonatal immunity to SARS-CoV-2 and protection from adverse outcomes is scarce. This work aimed to describe neonatal seropositivity in the context of maternal SARS-CoV-2 infection, seropositivity, and neonatal outcomes. This retrospective nested case-control study enrolled high-risk pregnant women with a SARS-CoV-2 RT-PCR positive test who gave birth at the Instituto Nacional de Perinatología in Mexico City and their term neonates. Anti-SARS-CoV-2 IgG antibodies in maternal and cord blood samples were detected using a chemiluminescent assay. In total, 63 mother-neonate dyads (mean gestational age 38.4 weeks) were included. Transplacental transfer of SARS-CoV-2 IgG occurred in 76% of neonates from seropositive mothers. A positive association between maternal IgG levels and Cycle threshold (Ct) values of RT-qPCR test for SARS-CoV-2 with neonatal IgG levels was observed. Regarding neonatal outcomes, most seropositive neonates did not require any mechanical ventilation, and none developed any respiratory morbidity (either in the COVID-19 positive or negative groups) compared to 7 seronegative neonates. Furthermore, the odds of neonatal respiratory morbidity exhibited a tendency to decrease when neonatal IgG levels increase. These results add further evidence suggesting passive IgG transfer importance.

## Introduction

Pregnant women with severe acute respiratory syndrome coronavirus 2 (SARS-CoV-2) have an increased risk of complications compared to non-pregnant women (1); those complications include neonatal adverse outcomes as stillbirth and preterm birth. International concerns rely on the possible infection risk in the unborn child or neonate, highlighting the impact of the humoral transferred immunity from mother to child before birth and during the lactation period. Neonates depend on the transfer of maternal antibodies across the placenta for protection from infections, particularly IgG (2). The efficiency of antibodies transference across the placenta is determined by different factors such as placental pathologies, maternal hypergammaglobulinemia, and the timing of mother infection and associated pathogen (2). Therefore, transplacental transfer of antibodies against the novel severe acute respiratory Coronavirus 2 (SARS-CoV-2) during COVID-19 maternal infection is of utmost importance for infant immunity.

The presence of IgG anti-SARS-CoV-2 antibodies in cord blood has been demonstrated (3–7), and it has been proposed that antibody transfer from woman to child relies on maternal antibody concentration and timing of maternal infection as in other infectious diseases (4, 8, 9). When maternal infection occurs during the third trimester of pregnancy, IgG transfer seems inefficient (6–10), leaving neonates at risk of infection (10). A reduced transplacental transfer of specific SARS-CoV-2 antibodies has been demonstrated (3, 10, 11). Quantitation of IgG titers revealed that only 75% of asymptomatic or mild disease pregnant women and one-third of their offspring developed specific SARS-CoV-2 antibodies at delivery moment consistent with the timing of maternal infection (14 days before delivery) (3).

However, the characterization of maternal-neonatal transfer of IgG anti-SARS-CoV-2 antibodies and neonatal outcomes remains limited. More information on passive maternal immunity and neonatal morbidity is urgently needed to develop strategies for infant protection and vaccine campaigns. This study aimed to describe neonatal seropositivity in the context of SARS-CoV-2 maternal immunity in an ongoing infection as well as neonatal outcomes in term deliveries. The following questions were tackled: Can we predict the mother-to-child transfer rate before delivery? Can neonatal adverse outcomes be improved when specific anti-SARS-CoV-2 antibodies are passively transferred from infected mothers? This work was performed during the pandemic peak in Mexico City; during this period, the prevalence of COVID-19 among pregnant women admitted to this healthcare center was 26–28% (12, 13). The research objectives of this study were therefore to determine the transplacental transfer rate of maternal antibodies against SARS-CoV-2 in COVID-19 infected pregnant women and to evaluate the rate of neonatal outcomes relative to serology status in term high-risk pregnancies.

## Methods

### Study Population and Ethical Approval

This retrospective nested control-case study was approved by the Institutional Review Boards of the National Institute of Perinatology (Instituto Nacional de Perinatología, INPer), the Research Committee and Ethics in Research Committee (grant numbers: 2020-1-32 and 2020-1-31). The study followed the principles of the Declaration of Helsinki and was performed according to the STROBE guidelines. Patients gave their written consent to participate and were fully anonymized. The study was conducted at the INPer, a third-level healthcare center attending only high-risk pregnancies. It is a national referral center with 3,500–4,000 pregnancies each year.

Pregnant women with laboratory-confirmed SARS-CoV-2 infection with a positive test result 24–48h before delivery by quantitative reverse transcription-polymerase chain reaction (RT-qPCR) in oropharyngeal or nasopharyngeal swabs, between March 2020 and January 2021, together with their term newborns were included. Neonates' inclusion criteria were at least one RT-qPCR test result at 12–48 h after birth and gestational age > 37 weeks. No further PCR tests were performed. Due to Mexican government regulations at recruitment time, none of the women were vaccinated. Phylogenetic Assignment of Named Global Outbreak (PANGO) revealed that dominant lineages in Mexico were B.1.1.519 (37.8%), B.1 (13.9%), B.1.1.222 (10.3%), B.1.1 (5.7%), B.1.609 (5.6%), and B.1.243 (4.5 %) being the most prevalent (14). In this large third-level healthcare center, universal screening of pregnant women was performed, and SARS-CoV-2 infection was assessed using the Cycle threshold (Ct) values obtained from the RT-qPCR assays, following La Charité Protocol (15). A Ct value <38 was defined as a positive result. Clinical, demographic, and outcome data were obtained from electronic medical records. Peripheral maternal blood and cord blood samples were collected immediately after delivery from all patients. Maternal variables included age, pre-gestational BMI, pre-existing conditions (hypertension, autoimmune disease, diabetes mellitus, hypothyroidism), gravity, C-section, abortion, and outcomes (gestational diabetes, preeclampsia, and premature rupture of membranes). The mode of delivery was C-section for all women. Birth outcomes evaluated were Apgar score at 1 and 5 min, ventilation at birth and during hospitalization [including oxygen, Continuous positive airway pressure (CPAP), invasive mechanical ventilation (IMV), endotracheal intubation (ETI), high-frequency ventilation (HFV)], respiratory morbidity [transitory tachypnea, respiratory distress (RDS), pneumothorax], number of hospitalization days, and destination at discharge (home or death). Apgar score classification recorded at 1 and 5 min is based on a total score of 10 and includes color, heart rate, reflexes, muscle tone, and respiration. A score of >7 is reassuring, 4–6 moderately abnormal, and 0–3 low (16).

### Clinical Classification of Neonatal/Pediatric Infection

COVID-19 severity was classified according to (17, 18), based on clinical, radiological, and laboratory features, as follows:

#### Asymptomatic Infection

Patients with no clinical or radiological manifestations, but a SARS-CoV2 positive test result by RT-qPCR.

#### Mild Infection

Patients present respiratory or gastrointestinal symptoms, including fever, cough, sneezing, diarrhea, and vomiting.

#### Moderate Infection

Patients have features of viral bronchitis and pneumonia with no tachypnea and hypoxemia.

#### Severe Infection

Patients have tachypnea and hypoxemia with SaO2 <92% in room air.

### SARS-CoV-2 IgG Antibodies Assay

A chemiluminescent immunoassay (SARS-CoV-2 IgG II Quant, 6SR86) was performed to detect anti-SARS-CoV-2 IgG antibodies against the nucleocapsid protein in maternal and cord blood samples (19) (Abbot Laboratories, Abbot Park, IL, United States). Maternal peripheral blood was obtained at delivery. In this method, the amount of IgG antibodies to SARS-CoV-2 in each sample was determined by comparing its chemiluminescent relative light unit (RLU) to the calibrator or control RLU (index S/C). The assay reports an index value based on the ratio of sample absorbance to the absorbance of an assay-specific calibrator or control (S/C). A positive result for anti-SARS-CoV-2 antibodies is denoted by a cut-off index >1.4 (sample to control index, s/co). Using this cut-off index, the manufacturer reported a sensitivity of 86·4% after 7 days from symptom onset and 100% after 14 days, and a specificity of 99·6%, using RT-PCR as the gold standard. All tests were performed in duplicate according to the manufacturer's instructions.

### Statistical Analysis

Descriptive statistics were evaluated, medians and Interquartile range (IQR) were reported for numerical values; numbers and percentages with 95% Confidence Intervals (CIs) were reported for categorical variables. Correlations were evaluated using Spearman's correlation. All cut-off indexes were Log2-transformed. A stepwise logistic regression model was fit to evaluate which of the following maternal variables (Ct values, SARS-CoV-2 IgG Index) were most likely to be associated with a cord blood seropositive result. Then, the logistic regression model was performed considering maternal age, pre-gestational overweight/obesity, and pre-existing condition (diabetes mellitus). Next, in order to assess the association of anti-SARS-CoV-2 antibody index with neonatal morbidity (outcome), a logistic regression model was fit (adjusted for neonatal variables PCR test result, sex, and reanimation at birth). The model was a step-by-step iterative construction that selects independent variables (predictors) and tests for significance after each iteration to build the best-performing model. We started with no predictors in the regression equation in the first step. Then, to control how the variables were included in the regression, the Enter method was used, in which all independent variables in a block were entered into the regression analysis in a single step (variables must pass tolerance tests to be included). The omnibus Test of Model Coefficients result was *P* < 0.05 (validity of the models). Statistical significance was set at *P* < 0.05. Analyses were performed using SPSS version 27 (IBM Corp., Armonk, N.Y., United States).

## Results

### Cord Blood SARS-CoV-2 Seropositivity: Maternal Correlation

**Figure 1:**
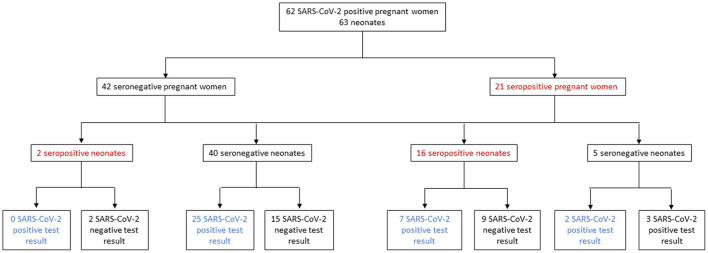
Pregnant women and neonates included in the study.

Sixty-two pregnant women with confirmed SARS-CoV-2 infection by RT-qPCR at delivery and 63 term neonates (one twin pregnancy) were included in this study, of whom 29 (46%) neonates had a SARS-CoV-2 positive RT-qPCR test. SARS-CoV-2 specific IgG antibodies in maternal and cord blood samples were measured to investigate transplacental transfer. 21 women (34%, 95% CI, 22%−45%) were seropositive at delivery. Among neonates born to seropositive women, 16 tested positive for anti-SARS-CoV-2 IgG antibodies in cord blood (76%, 95% CI 55−90%), of whom 7 and 9 had a positive and negative test result for SARS-CoV-2 by RT-qPCR (Figure 1). The baseline characteristics across the mother-neonate dyads are shown in Table 1 (Supplementary Material). All pregnant women were asymptomatic except one who presented mild symptoms (cough and rhinorrhea). Regarding conditions that have been associated with the severity of COVID-19, 34% of women were older than 35 years, and 33% had pre-gestational obesity. One woman had hypertension, two women had autoimmune diseases, and 4 had diabetes mellitus (Table 1, Supplementary Material). Adverse outcomes observed were 2 cases of preeclampsia, 2 cases of gestational diabetes, and 1 case of premature rupture of membranes (Table 1, Supplementary Material). The median gestational age (GA) at delivery was 38.7 weeks (IQR, 38.2−39.5 weeks), and neonatal birth weight was 3110 g (IQR, 2,790−3,345 g). Most neonates had a birth weight > 2,500 g (94%).

**Table 1:** Neonatal outcomes relative to seropositivity in cord blood samples in the COVID-19 positive group.

	**COVID-19 positive (*****n*** **=** **34)**	
	**Seronegative cord blood (*n* = 27)**	**Seropositive cord blood (*n* = 7)**
**Classification of COVID-19 infection:** Asymptomatic Mild Moderate Severe	23 (100) 0 2 (7) 2 (7)	7 (100) 0 0 0
**1 min Apgar score:** 4–6 7–9	0 27 (100)	0 7 (100)
**5 min Apgar score:** 4–6	27 (100)	7 (100)
Reanimation at birth: None Oxygen ETI	13 (50) 12 (46) 1 (4)	3 (43) 4 (57) 0
**Ventilatory support:** None Oxygen CPAP HFV	22 (81) 3 (11) 2 (7) 0	7 (100) 0 0 0
**Respiratory morbidity:** None Tachypnea RDS	25 (93) 2 (7) 0	7 (100) 0 0
**Hospital stay:** 1–3 4–7 7–14 >14	19 (73) 3 (11) 3 (11) 1 (4)	5 (71) 1 (14) 0 1 (14)
**Final outcome:** Home Death	27 (100) 0	11 (100) 0

*CPAP, continuous positive airway pressure; ETI, endotracheal intubation; IMV, invasive mechanical ventilation; HFV, High frequency ventilation; RDS, respiratory distress*.

**Figure 2:**
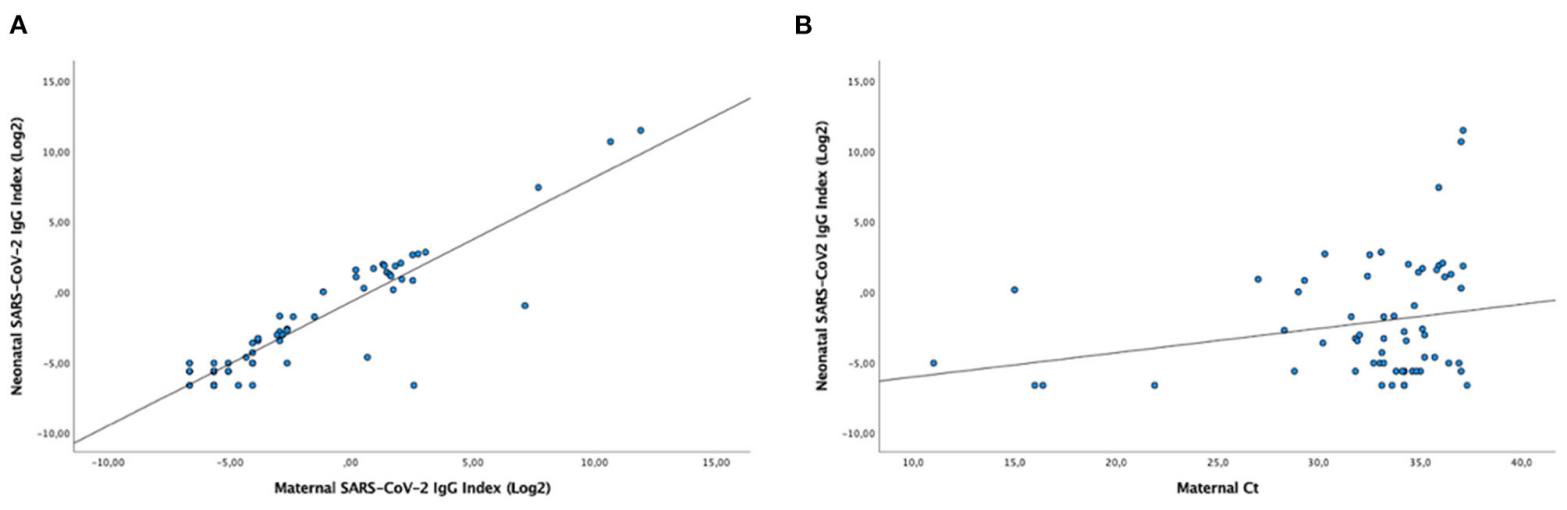
T Correlation between cord blood seropositivity and maternal seropositivity **(A)** or cord blood seropositivity and maternal Ct values at delivery **(B)**. Scatterplots show the distribution of SARS-CoV-2 IgG levels as an index value based on the ratio of sample absorbance to the absorbance of an assay-specific calibrator or control (S/C) units at the time of delivery. (R = 0.851, *P* <0.0001 and R = 0.228, *P* = 0.075, for a and b, respectively; Pearson Correlation).

In the RT-qPCR test, the median maternal and neonatal Ct values were 34.2 (IQR, 31.8−35.1) and 25.9 (IQR, 30.5−34.7), respectively. The cord and maternal serum SARS-CoV-2 IgG index at delivery were strongly positively correlated (R = 0.851, *P* < 0.0001) (Figure 2A). There was a tendency for a positive correlation between cord blood IgG index and maternal Ct values (R = 0.228, *P* = 0.075) (Figure 2B).

Next, forward logistic regression analyses were fit to assess the association of seropositivity in cord blood samples with maternal Ct values and SARS-CoV-2 IgG index. The model was statistically significant (*P* < 0.001, Nagelkerke R2 = 0.748), correctly predicted 95.2% of cases, and showed that increasing maternal SARS-CoV-2 IgG index was significantly associated with an increased likelihood of having cord blood seropositivity and a trend for Ct values, compared to non-seropositive samples (Table 2, Supplementary Material). We then performed a forward logistic regression analysis to ascertain the effects of maternal age, pre-gestational BMI, and diabetes mellitus, but none of these pre-existing conditions (associated with severity of COVID-19 disease) add significantly to the model (*P* = 0.832, *P* = 0.990, and *P* = 0.996, for maternal age, pre-gestational BMI, and diabetes mellitus, respectively; data not shown).

**Table 2:** Neonatal outcomes relative to seropositivity in cord blood samples in the COVID-19 negative group.

	**COVID-19 negative (*****n*** **=** **29)**	
	**Seronegative cord blood (*n* = 18)**	**Seropositive cord blood (*n* = 11)**
**1 min Apgar score:** 4–6 7–9	1 (6) 17 (94)	0 11 (100)
**5 min Apgar score:** 4–6	18 (100)	11 (100)
**Reanimation at birth:** None Oxygen ETI	11 (61) 7 (39) 0	7 (64) 4 (36) 0
**Ventilatory support:** None Oxygen CPAP HFV	16 (89) 0 1 (6) 1 (6)	10 (91) 0 1 (9) 0
**Respiratory morbidity:** None Tachypnea RDS	16 (89) 1 (6) 1 (6)	11 (100) 0 0
**Hospital stay:** 1–3 4–7 7–14 >14	16 (94) 1(6) 0 0	9 (82) 1 (9) 1 (9) 0
**Final outcome:** Home Death	16 (94) 2 (12)	11 (100) 0

*CPAP, continuous positive airway pressure; ETI, endotracheal intubation; IMV, invasive mechanical ventilation; HFV, High frequency ventilation; RDS, respiratory distress*.

### Cord Blood SARS-CoV-2 Seropositivity: Neonatal Outcomes

Independently of the IgG status, ventilation requirements, and length of hospital stay were similar among COVID-19 positive and negative neonates; most neonates in these groups did not require any ventilation support during hospitalization (Table 3, Supplementary Material). We then assessed neonatal outcomes between seronegative and seropositive neonates in the COVID-19 positive and negative groups (Table 1 and Table 2, respectively). Among the 34 COVID-19 positive neonates, 30 were asymptomatic (86%) while 4 were symptomatic (14%); of whom 2 had moderate disease, and 2 had severe disease (Table 1).

In the COVID-19 positive group (Table 1), 27 neonates were seropositive, and 7 were seronegative. All seropositive neonates were asymptomatic (*n* = 7) compared to 4 symptomatic seronegative neonates, classified as moderate or severe disease. Regarding ventilatory support during hospitalization, among seronegative neonates, 3 required oxygen therapy and 2 continuous positive airway pressure (CPAP) compared to none of the seropositive neonates. Seven seronegative and 2 seropositive neonates were hospitalized > 4 days.

Among infants with a COVID-19 negative test (Table 2), 1 seropositive neonate required ventilation (CPAP) compared with 2 seronegative neonates on respiratory support at birth with FiO_2_ in the range of 20–35%; of whom 1 on CPAP and 1 with High-Frequency Ventilation (HFV) due to RDS. No seropositive infants developed respiratory morbidity compared with 2 seronegative neonates that presented tachypnea and RDS. We found 2 deaths in the seronegative group; one of them had cardiomyopathy and died 24 h after birth, probably of unrelated COVID-19 disease. The second one developed RDS, was on HFV support, and died of septic shock at birth. Both neonates tested negative for SARS-CoV-2 the day of birth (<12 h), and no other PCR tests could be obtained.

Next, to assess if there was an association between neonatal SARS-CoV-2 antibody index with the odds of having any respiratory morbidity (yes or no), a logistic regression model was fit, controlled for neonatal risk factors (PCR test result, reanimation at birth (yes/no), and sex). The model was statistically significant (*P* = 0.001, Nagelkerke R2 = 0.685) and correctly predicted 95.1% of cases. The odds of neonatal respiratory morbidity vs. no morbidity exhibited a tendency to decrease when neonatal IgG levels increase by a factor of 0.284 (*P* = 0.057; Table 4, Supplementary Material).

Taken together, these results suggest fewer seropositive infants with respiratory morbidity compared to seronegative infants, although the majority of infants were asymptomatic.

## Discussion

Our study conducted a retrospective observational study in 63 mother-neonate dyads in pregnant women infected with SARS-CoV-2 to understand better the association between maternal IgG presence and passive immunity. All 62 mothers had a positive RT-PCR test result for SARS-CoV-2 at delivery. However, they presented a low rate of positive sera; only 34% of women possessed specific SARS-CoV2- IgG antibodies. It is essential to mention that most women were asymptomatic (61/62 women, 98.6%), as found previously in this health care center (12, 13). This study showed that 76% of neonates from seropositive mothers had antibodies against SARS-CoV-2 in cord blood, of whom 56% had a SARS-CoV-2 negative test result at birth. Neonatal SARS-CoV-2 antibodies index was positively associated with maternal anti-SARS-CoV-2 IgG levels and Ct values. Regarding neonatal outcomes, most seropositive neonates did not require any ventilation, and none developed any respiratory morbidity either in the COVID-19 positive or negative groups. In contrast, 7 seronegative neonates required ventilation during hospitalization, of whom 4 neonates were classified as a moderate and severe COVID-19 disease.

In this study, only 34% of pregnant women were seroconverted. Concerning seropositivity, previous studies reported higher rates of SARS-CoV-2 IgG antibodies in pregnant women (from 60% to 100%) compared to this work (9, 20), but most women were symptomatic mothers. For asymptomatic or with mild disease, seroconversion occurs late or remains negative (21, 22); this fact is interesting as seroconversion is essential in pregnant women's passive immunity. In that sense, cases of RT-PCR positive mothers without specific IgG might be due to an early infection or lack of seroconversion. Indeed, IgG seroconversion has been shown to occur 8 days after symptom onset and increase to 100% after 19 days (23). It has been reported that asymptomatic patients have a lower immune response to SARS-CoV-2 (24). It has been proposed that the differences in antibody titers and disease severity might be due to excessive inflammation and/or uncontrolled SARS-CoV-2 replication found during severe COVID-19. Also, viral load has an implication, as initial amounts of viral antigens may contribute to more robust serological responses (25). Accordingly, our studied population was asymptomatic and possessed lower viral loads, possibly leading to a decreased seroconversion rate. However, we showed that SARS-CoV-2 specific IgG antibodies were transferred to 76% of neonates from their seropositive mothers, suggesting placental transferal of immunity. Lack of antibodies in 24% of neonates from seropositive mothers rely on two main possibilities: the timing of infection in the mother; in that sense, it might be possible that antibodies were new and there was no time for the transfer. The other possibility might be the decreased placental antibody transfer demonstrated in pregnant women infected with SARS-CoV-2 during the second (4, 26–28) or third trimester of pregnancy (6) and in ongoing infection compared to recovered or uninfected mothers (4, 26, 28, 29). Serological tests are essential for vulnerable populations such as pregnant women because immune status has implications for managing pregnant women and newborns (20). But as in other viral infections, it is also proposed that maternal immune system activation by SARS-CoV-2 might impact the newborn's health and immune system development (29, 30). Recently, immune imprinting of the neonate in mothers with a SARS-CoV-2 infection has been shown with an imbalance favoring pro-inflammatory responses (4, 28, 29, 31). Further characterization of seropositive and seronegative neonate's immune response from our study is warranted.

A positive correlation between fetal and maternal IgG titers was found in this work; this phenomenon was described previously in mothers infected during pregnancy and in vaccinated ones (4, 25, 27, 28). We demonstrated a positive association between maternal IgG titers and antibody transfer (seropositivity in cord blood), confirming that passive antibody transfer not only depends on the trimester of infection but also on the robust maternal antibody response, as previously suggested (6). Together with maternal IgG titers, we used Ct values to predict seropositivity in neonates, and the model showed that using both variables (IgG titers and Ct value), it is possible to predict 92% of the neonatal seropositive cases. This could be helpful for neonatal management, mostly when mothers are not vaccinated.

This study showed that 53.5% of neonates tested positive for SARS-CoV-2 during the first 12–48 h after delivery, 11% were seropositive and 42% were seronegative. In previous works, we have demonstrated viral presence in fetal tissue (32), in placentas from positive mothers (33, 34), and in amniotic fluid and newborns (saliva and rectal swabs) at delivery, even in the presence of maternal IgG (33, 34). The results shown in the present work do not allow us to conclude about viral transmission, as only one single swab sample was obtained of most neonates. Nevertheless, as neonatal swabs were collected at 12–48 hrs after birth, viral transmission might occur by vertical transmission. According to the Definition and categorization of the timing of mother-to-child transmission of SARS-CoV-2 by the WHO classification (35), vertical transmission can occur by three mechanisms: *in utero* transmission (A), intrapartum transmission (B), and early postnatal transmission (C). (A) *in utero* transmission in a live birth must show: (1) evidence of maternal SARS-CoV-2 infection anytime during pregnancy, and (2) *in utero* fetal exposure, and (3) SARS-CoV-2 persistence or immune response in the neonate (B). Intrapartum transmission requires (1) evidence of maternal SARS-CoV-2 infection near the time of birth, and (2) evidence of lack of *in utero* fetal SARS-CoV-2 exposure (RT-PCR negative test result <24 h), and (3) intrapartum exposure with viral persistence or immune response in the infant (evidence <48 h) (C). Early postnatal transmission requires (1) evidence of maternal SARS-CoV-2 infection near the time of birth, and (2) evidence of lack of *in utero* fetal SARS-CoV-2 exposure (RT-PCR negative test result <48 h), and (3) early postnatal exposure with viral persistence at >48 h.

Our results demonstrate two possible transmission mechanisms. In some cases, we have evidence of (1) maternal infection before birth, (2) *in utero* exposure (neonatal RT-PCR <24 h), but we do not have evidence of persistence of infection. Therefore, they fit into the WHO classification “indeterminate *in utero* transmission”. In other cases, we have evidence of (1) maternal infection before birth, (2) lack of evidence of *in utero* exposure (neonatal RT-PCR <24h not performed), but we do have evidence of persistence of infection (RT-PCR at 24 to 48 h); fitting into the WHO classification as “Possible intrapartum transmission”. According to our results and the WHO classification, our neonates acquired SARS-CoV-2 infection by vertical transmission (*in utero* or early postnatal contact).

This study found a diminished number of seropositive neonates with respiratory morbidities compared to seronegative neonates. As well, we observed a trend in predicting respiratory morbidity by the concentration of neonatal SARS-CoV-2 antibodies. Collectively, these results add further information to the ongoing but limited knowledge of the association between the transplacental transfer of SARS-CoV-2 antibodies with neonatal outcomes. This is a priority for future research related to the transfer of maternal immunity to the infant (36, 37). This information is ultimately aimed to help health policymakers and is of clinical relevance.

### Limitations of the Study

Several limitations must be acknowledged. First, the number of patients is relatively small from one health care center; however, this third-level center is a large referral center for pregnancies in Mexico. Second, most women admitted for delivery were asymptomatic (98.6%); thus, the results may not be compared to the symptomatic pregnant population. Third, since the onset of exposure or infection cannot be determined in asymptomatic women and the number of days between the beginning of the disease and the SARS-CoV-2 test at delivery was unknown, we could not compare the results considering the timing of symptoms. Thus, even with ongoing infection, this could impact the Ct values and IgG SARS-CoV-2 titers between pregnant women, and this data could not be considered. Fourth, only term high-risk pregnancies were evaluated. This could underestimate the frequency of adverse outcomes since premature infants were not selected and affect the generalizability of the study results in preterm newborns.

To date, current evidence is not conclusive that IgG transfer from mothers to their offspring confers SARS-CoV-2 immunity, but it could be crucial to reduce neonatal adverse outcomes. These results add further evidence suggesting maternal IgG transfer importance. Studies on larger populations are urgent, but after almost 2 years of pandemic and with the knowledge generated about immunity and neonatal outcomes, our study suggests that SARS-CoV-2 transplacental transfer during pregnancy is important for newborns health.

## Data Availability Statement

The raw data supporting the conclusions of this article will be made available by the authors, without undue reservation.

## Ethics Statement

The studies involving human participants were reviewed and approved by Research Committee and Ethics in Research Committee of the Instituto Nacional de Perinatología. The patients/participants provided their written informed consent to participate in this study.

## Author Contributions

Conceptualization: AH-R, JC-P, and CI. Methodology: AH-R, GA-P, IV-M, ML-J, MV-V, GM-S, MC-G, and CC-J. Validation: IC-Z, MR-R, MR-B, GL-N, and SA-G. Formal analysis: AH-R and CI. Investigation: GA-P, IV-M, ML-J, MR-R, GA-R, PM-R, GL-N, SA-G, MC-G, EM-V, CC-J, and GM-S. Resources: JC-P, MC-B, MC-G, and CC-J. Data curation: AH-R, SE-y-S, and CI. Writing—original draft preparation: AH-R and C.I. Writing—review and editing: AH-R, GL-N, MR-R, and CI. Supervision: AH-R, JC-P, MC-B, and CI. Project administration: JC-P and CI. Funding acquisition: JC-P, MC-B, MC-G, and CC-J. All authors have read and agreed to the published version of the manuscript. All authors contributed to the article and approved the submitted version.

## Funding

This research was funded by the INSTITUTO NACIONAL DE PERINATOLOGÍA, grant numbers 2020-1-31, 2020-1-32, and 2021-1-31.

## Conflict of Interest

The authors declare that the research was conducted in the absence of any commercial or financial relationships that could be construed as a potential conflict of interest.

## Publisher's Note

All claims expressed in this article are solely those of the authors and do not necessarily represent those of their affiliated organizations, or those of the publisher, the editors and the reviewers. Any product that may be evaluated in this article, or claim that may be made by its manufacturer, is not guaranteed or endorsed by the publisher.
