# Supplementary material for: Cord Blood SARS-CoV-2 IgG Antibodies and Their Association With Maternal Immunity and Neonatal Outcomes
Source: Front Pediatr. 2022 Jun 29;10:883185. doi: 10.3389/fped.2022.883185 (PMC9277091; doi:10.3389/fped.2022.883185)
Supplement: Supplementary file 1 [file Table_1.DOCX]

Supplementary Material

# Supplementary Tables

**Table 1**. Clinical characteristics of the maternal-neonatal dyads.

|  | **Seronegative cord blood**  **(n=45)**  **(n, %)** | **Seropositive cord blood**  **(n=18)**  **(n, %)** | **All**  **(n= 63)**  **(n,%)** |
| --- | --- | --- | --- |
| **Maternal age (years):**  **<19**  **19-34**  **>35** | 1 (2)  26 (58)  14 (22) | 1 (5)  11 (61)  6 (33) | 2 (3)  37 (63)  20 (34) |
| **Maternal pre-gestational BMI (kg/m2):**  **<24.9**  **25-29.9**  **>30** | 3 (8)  23 (60)  12 (32) | 3 (18)  8 (47)  6 (33) | 6 (11)  31 (56)  18 (33) |
| **Maternal pre-existing conditions:**  **Hypertension**  **Autoimmune disease**  **Diabetes Mellitus**  **Hypothyroidism** | 0  2 (5)  4 (7)  2 (4) | 1 (5)  0  0  0 | 1 (1)  2 (3)  4 (6)  2 (3) |
| **Gravity:**  **1**  **2**  **>3** | 13 (29)  13 (29)  14 (31) | 3 (17)  10 (55)  3 (17) | 16 (29)  23 (41)  17 (30) |
| **C-section:**  **0**  **1**  **2**  **>3** | 20 (44)  16 (35)  6 (13)  3 (17) | 9 (50)  5 (28)  2 (11)  0 | 29 (52)  16 (29)  8 (14)  3 (5) |
| **Abortion:**  **0**  **1**  **>2** | 24 (50)  13 (29)  3 (67) | 10 (55)  4 (22)  2 (11) | 34 (61)  17 (30)  5 (9) |
| **Gestational Diabetes** | 1 (2) | 1 (6) | 1 (1) |
| **Preeclampsia** | 0 | 2 (11) | 2 (3) |
| **Premature rupture of membranes** | 1 (2) | 0 | 1 (1) |
| **Gestational age (weeks)**  **> 37** | 45 (100) | 18 (100) | 63 (100) |
| **Birthweight (g):**  **1501-2499**  **>2500** | 4 (9)  41 (91) | 0  18 (100) | 4 (6)  59 (94) |

**Table 2.** A binomial logistic regression model with cord blood antibody test result (positive vs. negative) as a dependent variable.

| **Variables** | **B** | **Wald** | **Sig.** | **Exp (B)** | **95% CI for Exp (B)** |
| --- | --- | --- | --- | --- | --- |
| Maternal Log2 SARS-CoV-2 IgG index | 0.857 | 13.756 | <0.001 | 2.357 | 1.498 – 3.707 |
| Maternal Ct values | 0.213 | 3.340 | 0.068 | 1.238 | 0.985 – 1.555 |

**Table 3**. Neonatal outcomes according to the RT-qPCR test result.

|  | **COVID-19 negative**  **(n=29)** | | **COVID-19 positive**  **(n=34)** | |
| --- | --- | --- | --- | --- |
|  | **Seronegative cord blood**  **(n=18)** | **Seropositive cord blood**  **(n=11)** | **Seronegative cord blood**  **(n=27)** | **Seropositive cord blood**  **(n=7)** |
| **Reanimation:**  **None**  **Oxygen**  **ETI** | 10 (59)  7 (41)  0 | 7 (64)  4 (36)  0 | 13 (50)  12 (46)  1 (4) | 3 (43)  4 (57)  0 |
| **Ventilatory support:**  **None**  **Oxygen**  **CPAP**  **HFV** | 16 (94)  0  0  1 (6) | 10 (91)  0  1 (9)  0 | 22 (81)  3 (11)  2 (7)  0 | 7 (100)  0  0  0 |
| **Respiratory morbidity:**  **None**  **Tachypnea**  **RDS** | 15 (88)  1 (6)  1 (6) | 11 (100)  0  0 | 25 (93)  1 (4)  0 | 7 (100)  0  0 |
| **1 min Apgar score:**  **4-6**  **7-9** | 1 (6)  16 (94) | 0  11 (100) | 0  27 (100) | 0  7 (100) |
| **5 min Apgar score:**  **4-6** | 17 (100) | 11 (100) | 27 (100) | 7 (100) |
| **Lemgh of hospital stay:**  **1-3**  **4-7**  **7-14**  **>14** | 15 (94)  1(6)  1 (6)  0 | 9 (82)  1 (4)  1 (4)  0 | 19 (73)  3 (73)  3 (11)  1 (4) | 5 (73)  1 (14)  0  1 (14) |

CPAP: continuous positive airway pressure; IMV: invasive mechanical ventilation; HFV: High frequency ventilation; RDS: respiratory distress

**Table 4.** A binomial logistic regression model with respiratory morbidity (adverse outcome) as a dependent variable and its association with cord blood antibody index.

| **Variables** | **B** | **Wald** | **Sig.** | **Exp (B)** | **95% CI for Exp (B)** |
| --- | --- | --- | --- | --- | --- |
| SARS-CoV-2 IgG index | -1.260 | 3.614 | 0.057 | 0.284 | 0.077 – 1.040 |
| PCR test result | -3.306 | 2.646 | 0.212 | 0.212 | 0 - 6.561 |
| Sex | 4.067 | 2.212 | 0.137 | 58.396 | 0.275 - 12419.2 |
| Reanimation at birth | 21.3 | 0 | 0.997 | 1.78 | 0 |
